# Appraisal of Minerals, Fasting Blood Glucose, and Lipid Parameters in Obese and Nonobese Premenopausal Women

**DOI:** 10.7759/cureus.60995

**Published:** 2024-05-24

**Authors:** Nandini Kondagadapu, Dawood Suleman, Sabitha Vadakedath, Venkataramana Kandi

**Affiliations:** 1 Biochemistry, Prathima Institute of Medical Sciences, Karimnagar, IND; 2 Biochemistry, Government Medical College & Hospital, Mancherial, Mancherial, IND; 3 Clinical Microbiology, Prathima Institute of Medical Sciences, Karimnagar, IND

**Keywords:** lipid parameters, obese women, fasting blood glucose (fbg), phosphorus, magnesium, calcium, minerals, premenopausal women

## Abstract

Introduction

Menopause is an important milestone in the lives of women. Despite it being a natural phenomenon, menopause brings a lot of changes in a woman's life, which significantly affects their health and well-being. Menopause involves the cessation of hormone production necessary for menstrual cycles and fertility of females. The absence of these hormones may disturb the homeostasis of minerals, blood glucose, and lipid parameters and predispose women to several health conditions affecting different organs. Obesity has been identified as one of the several conditions that influence the health of women. Therefore, assessing women's health before menopause may improve understanding of their well-being and predict problems during and after menopause. The present study evaluated the activities of calcium, magnesium, phosphorous, fasting blood glucose (FBG), and lipid parameters in obese and nonobese premenopausal women.

Methods

The present study included 90 obese and 110 nonobese premenopausal women attending the General Medicine and Obstetrics and Gynaecology Departments of Gandhi Medical College and Hospital (GMC&H), Secunderabad, Telangana, India. The body mass index (BMI) was measured in all the study participants to put them under obese and nonobese categories. Blood samples were collected from all the study participants for the estimation of the activities of minerals like calcium, magnesium, phosphorous, FBG, and lipid parameters including total cholesterol (TC), triglycerides (TG), very low-density lipoproteins (VLDL), low-density lipoproteins (LDL), and high-density lipoproteins (HDL).

Results

The results demonstrated a significant difference in the activities of lipid parameters (TC-obese (158.90 ± 20.20 mg/dl) versus nonobese (148.7 ± 18.6 mg/dl), p < 0.05; TG-obese (143.1 ± 58.2 mg/dl) versus nonobese (118.40 ± 55.80 mg/dl), p < 0.01; VLDL-obese (28.30 ± 11.50 mg/dl) versus nonobese (23.30 ± 11 mg/dl), p < 0.05; LDL-obese (92 ± 30.30 mg/dl) versus nonobese (73.90 ± 26.10 mg/dl), p < 0.01; HDL-obese (61.60 ± 12.50) versus nonobese (65.30 ± 11.25 mg/dl), p < 0.01), FBG (obese (106.80 ± 32.20 mg/dl) versus nonobese (88.50 ± 42.60 mg/dl); p < 0.01)), and magnesium (obese (1.79 ± 0.36 mg/dl) versus nonobese (2.42 ± 0.67 mg/dl); p < 0.01)). However, the activities of calcium (obese (9 ± 0.54 mg/dl) vs. nonobese (8.9 ± 0.58); p > 0.05)) and phosphorous (obese (3.84 ± 0.53 mg/dl) versus nonobese (3.75 ± 0.46 mg/dl); p > 0.05)) was found to be similar in obese and nonobese premenopausal women.

Conclusions

The results suggest that obese premenopausal women revealed lowered activities of magnesium that can predispose them to chronic diseases like cardiovascular diseases. In addition, obese women showed higher activities of FBG that predisposes them to type 2 diabetes mellitus (T2DM). There was significant variation in the lipid parameters among obese and nonobese women. However, serum calcium and phosphorous were similar in obese and nonobese premenopausal women.

## Introduction

Menopause is defined as the cessation of menstruation and menstrual cycles with the loss of fertility. It generally occurs in women over 45 years of age. Since menopause results in the stoppage of hormonal secretions, women develop many physiological changes. However, menopause does not set insidiously but women reach menopause over a period during which they experience menstrual cycle irregularities. In this period, women are described as belonging to the perimenopausal phase. Women who experience regular menstrual cycles and those who have not yet reached menopause and do not experience symptoms suggesting a perimenopausal phase are considered premenopausal women [1].

The menstrual cycles are essential to maintain the health of the ovaries, the uterus, and the female reproductive system [2]. Each menstrual cycle can be divided into phases based on events in the ovary (ovarian cycle) and the uterus (uterine cycle). The ovarian cycle consists of the follicular, ovulation, and luteal phases, whereas the uterine cycle is divided into menstruation, proliferative, and secretory phases [3]. The proliferative phase is the second phase of the uterine cycle when estrogen facilitates the growth of the uterine lining. As they mature, the ovarian follicles secrete increasing amounts of estradiol and estrogen. The estrogens initiate the formation of a new layer of endometrium in the uterus, histologically identified as the proliferative endometrium [4].

Although menopause is considered a natural process at the physiological level, there is a considerable decrease in the secretion of hormones like estrogen and progesterone at a very early age [1]. During the perimenopause phase, women experience variations in the duration of their menstrual cycles. The shorter cycles are due to reduced activities of estrogen and the long cycles are due to anovulation, where the eggs are not released from the ovaries. The hormonal changes during this phase predispose women to central adiposity. This is represented in women by less subcutaneous fat and increased total body fat, predisposing them to type 2 diabetes mellitus (T2DM) and cardiovascular diseases (CVDs) after menopause [5].

The transition to menopause in women may result in obesity, which is a chronic inflammatory state. Obesity may cause the release of adipokines from adipose tissue, which initiates an inflammatory cascade, resulting in insulin resistance and metabolic syndrome [6]. During the premenopausal phase, women may present with changes in total lipid content and blood glucose activities. Similarly, the activities of essential minerals also have an important role in physiological processes that support egg quality, maturation, implantation, and reduced oxidative stress [7].

Calcium is a macronutrient and a versatile intracellular messenger that plays an important role in bone and teeth formation. It influences vitamin D activities and helps in the secretion of hormones including ovarian, thyroid, insulin, and parathyroid hormone (PTH). Changes in the activities of calcium lead to insulin resistance. This is evident from the fact that menopause decreases bone density alters calcium levels and may result in insulin resistance [8].

Magnesium is a micronutrient that plays a key role in women’s reproductive function. The nutrient requirement varies during different phases of a woman’s life including childhood, puberty, pregnancy, and menopause [9]. Like calcium and magnesium, phosphorous is an essential mineral whose homeostasis depends on hormonal activities [10].

This study is carried out to measure the activities of minerals like calcium, magnesium, and phosphorus, fasting blood glucose (FBG), and lipid parameters in obese and nonobese premenopausal women.

## Materials and methods

This prospective, observational study included 200 premenopausal women (90 obese and 110 nonobese) aged between 30 and 50 years attending the General Medicine and Obstetrics and Gynaecology Departments of Gandhi Medical College and Hospital (GMC&H), Secunderabad, Telangana, India. The study was carried out between March 2016 and October 2017. The Institutional Ethics Committee of GMC&H approved (IEC/GMC/2016/24/11/2016) the study, and informed consent was obtained from all participants.

Inclusion and exclusion criteria

All premenopausal women, both obese and nonobese, who were healthy and had not been diagnosed with any comorbidities like hypertension and diabetes were included in the study. Pregnant women and those with comorbidities including thyroid abnormalities were excluded from the study.

The venous blood samples were collected from the study participants. The samples were centrifuged at 3000 revolutions per minute (rpm) for 10 minutes. The serum separated from the samples was used to estimate the activities of FBG, calcium, magnesium, phosphorus, and lipid parameters including total cholesterol (TC), triglycerides (TG), very low-density lipoproteins (VLDL), low-density lipoproteins (LDL), and high-density lipoproteins (HDL).

The study participants were divided into nonobese (18.5 kg/m^2 ^to <25 kg/m^2^) and obese (>30 kg/m^2^) based on the body mass index (BMI). FBG was estimated using the glucose oxidase-peroxidase (GOD-POD) method. Serum calcium, magnesium, and phosphorous were estimated using Arsenazo (1,8-dihydroxy-3,6-disulpho-2,7-naphthalene-bis(azo)-dibenzenearsonic acid), ultraviolet (UV)-phosphomolybdate, and xylidyl blue methods, respectively. TC was measured using the cholesterol oxidase (CHOD)-phenol-4-aminoantipyrine (PAP) method. Serum TG was measured using the glycerol phosphate oxidase (GPO) method. Randox Daytona plus analyzer (Randox Laboratories Ltd., Crumlin, United Kingdom) and ERBA XL 640 (Transasia Bio-Medicals Ltd., India) auto-analyzer were used to perform tests. The HDL, LDL, and VLDL cholesterol were estimated using the direct enzymatic method and Friedewald’s equation [11].

Statistical analysis

The data obtained were entered into a Microsoft Office 2019 Excel sheet (Microsoft® Corp., Redmond, WA). The IBM SPSS Statistics for Windows, Version 20 (released 2011; IBM Corp., Armonk, New York, United States) was used to draw statistical inferences, and the data were represented as percentages, mean, standard deviation, and p-value.

## Results

The mean age of the obese and nonobese study participants was 38.40 ± 5.41 years and 36.70 ± 5.34 years, respectively. The age-wise details of the study subjects are detailed in Table 1.

**Table 1:** Age-wise distribution of the study participants n: number, %: percentage

Age group (years)/participant category	Obese		Nonobese		Total n (%)
	n	%	n	%	
30-35	29	32.22	53	48.18	82 (41)
36-40	27	30	35	31.81	62 (31)
41-45	28	31.11	14	12.72	42 (21)
46-50	6	6.66	8	7.27	14 (7)
Total	90	100	110	100	200 (100)

The results demonstrated a significant difference in the activities of lipid parameters (TC-obese (158.90 ± 20.20 mg/dl) vs. nonobese (148.70 ± 18.60 mg/dl), p < 0.05; TG-obese (143.10 ± 58.20 mg/dl) vs. nonobese (118.40 ± 55.80 mg/dl), p < 0.01; VLDL-obese (28.30 ± 11.50 mg/dl) vs. nonobese (23.30 ± 11 mg/dl), p < 0.05; LDL-obese (92 ± 30.30 mg/dl) vs. nonobese (73.90 ± 26.10 mg/dl), p < 0.01; HDL-obese (61.60 ± 12.50 mg/dl) vs. nonobese (65.30 ± 11.25 mg/dl), p < 0.01), FBG (obese (106.80 ± 32.20 mg/dl) vs. nonobese (88.50 ± 42.60 mg/dl); p < 0.01)), and magnesium (obese (1.79 ± 0.36 mg/dl) vs. nonobese (2.42 ± 0.67 mg/dl); p < 0.01)) among obese and nonobese premenopausal women. However, the activities of calcium (obese (9 ± 0.54 mg/dl) vs. nonobese (8.9 ± 0.58 mg/dl); p > 0.05)) and phosphorous (obese (3.84 ± 0.53) vs. nonobese (3.75 ± 0.46 mg/dl); p > 0.05)) was found to be similar in obese and nonobese premenopausal women. The details of the activities of minerals, FBG, and lipid parameters among obese and nonobese premenopausal women are shown in Table 2.

**Table 2:** Comparison of minerals, FBG, and lipid parameters among the study participants #: statistically insignificant, p-value < 0.05: statistically significant, p-value < 0.01: statistically highly significant, n: number, SD: standard deviation, FBG: fasting blood glucose, VLDL: very low-density lipoproteins, LDL: low-density lipoproteins, HDL: high-density lipoproteins

Parameter	Obese (n = 90) Mean ± SD	Nonobese (n = 110) Mean ± SD	p-value
FBG (mg/dl)	106.80 ± 32.20	88.50 ± 42.60	<0.01
Magnesium (mg/dl)	1.79 ± 0.36	2.42 ± 0.67	<0.01
Calcium (mg/dl)	9 ± 0.54	8.90 ± 0.58	>0.05#
Phosphorus (mg/dl)	3.84 ± 0.53	3.75 ± 0.46	>0.05#
Total cholesterol (mg/dl)	158.90 ± 20.20	148.70 ± 18.60	<0.05
Triglycerides (mg/dl)	143.10 ± 58.20	118.40 ± 55.80	<0.01
HDL (mg/dl)	61.60 ± 12.50	65.30 ± 11.25	<0.01
VLDL (mg/dl)	28.30 ± 11.50	23.30 ± 11	<0.05
LDL (mg/dl)	92 ± 30.30	73.90 ± 26.10	<0.01

## Discussion

Women's transition to menopause causes a decline in the activities of estrogen. Estrogen contributes to an efficient defense system in women. Therefore, a drop in this hormone makes the conditions more conducive to inflammation mediated by various factors [12]. The molecular mechanism behind the action of estrogen on neurons indicates that the time, amount of hormone secretion, and the type of receptor used have a significant role in protecting the central nervous system (CNS) against neurotoxic stimuli [13]. Experimental research on mice showed that a decline in estrogen caused neuroinflammation by lowering intracellular magnesium levels [14].

Because of the role played by magnesium, calcium, and phosphorus in nerve and muscle function, variation in their activities impacts the functioning of nerves and muscles. The secretion of estrogen hormone increases calcium absorption by stimulating the enzyme alpha-1-hydroxylase present in the kidneys, either directly or indirectly. This is evident because the menopausal decline in estrogen secretions may decrease calcium absorption [15]. The calcium receptors of parathyroid cells sense the low blood calcium levels in menopausal women. The osteoclastic activity of PTH augments calcium levels by bone resorption [16].

In addition, the osteoclastic activity of PTH increases serum phosphorus levels. Results from previous studies revealed that in males, the age-related decline of phosphorus is continuous. Conversely, in females, after 46 years of age, there is a rise in serum phosphorus, independent of PTH, and dietary phosphorus intake. Furthermore, this rise in the activities of phosphorous was witnessed until 60 years of age. Thus, increased activities of phosphorous in menopausal women could predispose them to CVD [17,18]. In the present study, a decrease in the activities of serum calcium and phosphorus was slightly higher in nonobese women compared to obese premenopausal women.

Magnesium modulates nuclear factor kappa-B (NF-KB). NF-KB is a transcriptional factor that mediates inflammation [19-21]. This study’s findings show a decrease in the activities of magnesium in obese premenopausal women, indicating inflammation and oxidative stress. The oxidative stress impairs endothelial function due to the irreversible accumulation of oxidative products on the surface of the cells, leading to insulin resistance, hypertension, and dyslipidemia. Moreover, the present study results show moderate dyslipidemia and higher FBG in obese premenopausal women, which proves an inflammatory-induced metabolic disturbance during low magnesium conditions.

Magnesium plays an important role in intracellular insulin secretion by regulating the protein tyrosine kinase activity, insulin receptors, and peripheral insulin sensitivity [22]. Magnesium modulates the activities of enzymes involved in lipid metabolism like lipoprotein lipase (LPL), desaturase, 3-hydroxy-3-methyl-glutaryl-coenzyme A (HMG-CoA) reductase, and lecithin cholesteryl acyl transferase (LCAT). Furthermore, magnesium controls the levels of serum lipids and prevents dyslipidemia. Dyslipidemia seen in premenopausal women may be associated with reduced estrogen and a deficit in the modulation of lipid enzymes caused by magnesium [23]. The transition to menopause among women may contribute to hormonal fluctuations that lead to reduced activities of magnesium and calcium, along with high phosphorus levels. Therefore, it is necessary to understand the activities of minerals, FBG, and lipid parameters among women during premenopausal, perimenopausal, and postmenopausal stages. 

Not much literature is available regarding FBG, minerals, and lipid parameters among premenopausal women. More than a decade ago, a study similar to the present one was carried out among Iranian women. Results revealed that obese women had significantly lower serum magnesium (p = 0.035) and significantly higher FBG (p = 0.028) with notable variations in lipid parameters (p < 0.05) compared to nonobese premenopausal women [24]. Furthermore, a recent study from India demonstrated decreased serum calcium and magnesium activities among postmenopausal women compared to premenopausal women [25].

Study limitations

This study was carried out among a limited number of premenopausal women. The participants included in the study were not evaluated for their nutrition and dietary habits, which could have influenced the activities of minerals, FBG, and lipid parameters. Moreover, the study did not follow up with the study group toward peri- and postmenopausal stages, which could further increase the understanding of the changes in the parameters included and the resultant health complications.

## Conclusions

Menopause is a natural process associated with a decline in estrogen levels. The hormonal fluctuations during and after menopause predispose women to disorders in minerals like calcium, magnesium, and phosphorous. Because minerals play a key role in nerve conduction, the functioning of muscles, and hormone signaling, disturbances in their homeostasis impact women's health during premenopause, perimenopausal, and postmenopausal phases. Central adiposity is the major event occurring during the perimenopausal phase, which, if associated with obesity, contributes to metabolic disorders, dyslipidemia, T2DM, and CVD. Close monitoring of premenopausal women for blood parameters like FBG, minerals, and lipids could contribute to predicting potential health complications and taking preventive measures like physical exercises, diet modifications, and nutritional supplementation.
